# Videoconference-Delivered Cognitive Behavioral Therapy for Parents of Adolescents With Internet Addiction: Pilot Randomized Controlled Trial

**DOI:** 10.2196/60604

**Published:** 2024-10-03

**Authors:** Hideki Horita, Yoichi Seki, Takumi Yamaguchi, Yuki Shiko, Yohei Kawasaki, Eiji Shimizu

**Affiliations:** 1 Department of Cognitive Behavioral Physiology Graduate School of Medicine Chiba University Chiba City Japan; 2 Department of Occupational Therapy International University of Health and Welfare Narita City Japan; 3 Cognitive Behavioral Therapy Center Chiba University Hospital Chiba University Chiba City Japan; 4 Research Administration Center Saitama Medical University Saitama Japan; 5 Clinical Laboratory Department Chiba University Hospital Chiba University Chiba City Japan; 6 Preventive Medicine Center Chiba University Chiba City Japan

**Keywords:** internet addiction, adolescents, parents, cognitive behavioral therapy, digital health

## Abstract

**Background:**

The rise in internet addiction, including web-based gaming and social networking services, is a serious concern. Even with access to medical institutions and counseling services, individuals with internet addiction, particularly adolescents, often refuse medical treatment or counseling. Parent-focused psychological intervention may lead to positive outcomes by improving the parent-adolescent relationship and helping parents identify and modify their adolescent’s problematic behaviors, including internet addiction.

**Objective:**

This study was a pilot randomized controlled trial to test the feasibility of remote cognitive behavioral therapy via videoconferencing for parents of adolescents with internet addiction.

**Methods:**

A total of 13 parents of adolescents aged 12-20 years with internet addiction were recruited and randomly assigned to either 12 sessions of the videoconference-delivered cognitive behavioral therapy (vCBT) group (n=6, 46%) or the waitlist control group (n=7, 54%). The study period was from March 1, 2018, to March 31, 2022. The primary outcome was the scores of the Young Internet Addiction Test reported by the adolescents. The secondary outcomes were adolescents’ hours of internet use per day (Internet Addiction Test), reported by the adolescents and by their parents; the Young Diagnostic Questionnaire, completed by the parents; and the quality of life of the adolescents and the parents, measured by the EQ-5D-5L. These were evaluated at weeks 0 and 13.

**Results:**

As the primary outcome, the mean total Internet Addiction Test score decreased from 67.7 (SD 18.3; 6/13, 46%) at week 0 to 56.2 (SD 25.1; 5/9, 56%) at week 13 in the vCBT group, compared to an increase from 66.9 (SD 21.9; 7/13, 54%) to 68.0 (SD 18.7; 4/9, 44%) in the control group. For all outcomes, no significant differences were found between the 2 groups (all *P*>.05).

**Conclusions:**

This study suggested the practical feasibility of vCBT for parents of adolescents with internet addiction. Further large-scale, multicenter randomized controlled trials are necessary to examine the effectiveness.

**Trial Registration:**

UMIN Clinical Trials Registry UMIN000032483; https://tinyurl.com/yuhen6c9

## Introduction

The invention of the internet changed the world in unimaginable ways. With the internet, instantaneous and comprehensive exchange of large amounts of information with the rest of the world is possible. In the information age, the internet directly and indirectly impacts various aspects of people’s lives; it is used, among other things, for information gathering, entertainment, communication, and buying and selling products and services [1]. However, with its continual development, its negative aspects, such as fraud, crime, cyberbullying, and wasteful spending, have come to the front; the problem of internet addiction is one such issue that has received considerable scholarly attention [2-5].

Research on internet addiction has been ongoing since the 1990s; studies have aimed to address the serious consequences associated with growing internet use, such as low grades, withdrawal, disordered eating habits, and lack of sleep [6-8]. Internet addiction can lead to depression, aggression, exacerbation of general psychiatric symptoms, and a decline in self-esteem, which can affect a person’s career prospects and social support [9-15]. However, while studies have highlighted the importance of providing ongoing support for individuals with internet addiction, the number of medical and educational institutions that provide support remains limited [16-18]. In addition, even when a parent or guardian (hereafter referred to as a parent) of a person with internet addiction has access to medical institutions and counseling services to overcome the same, individuals with the addiction often refuse treatment, leaving them struggling with the addiction. In many cases, parents lack continuous access to medical institutions and counseling services; thus, the entire family is unable to receive the necessary support.

Parental factors have a significant impact on their children’s risk of developing internet addiction [19,20]. In addition, many adolescents with internet addiction may be reluctant to receive treatment or have difficulty attending the intervention sessions. Therefore, researchers recommend designing interventions focusing on parents. Such parent-focused interventions can lead to positive outcomes for the entire family by improving the parent-child relationship and helping parents identify and modify their child’s problematic behaviors using evidence-based methods.

Cognitive behavioral therapy (CBT) is a psychotherapy that helps thoughts and behaviors that have become rigid and narrow due to emotional stress to be more flexible and open. It is effective in treating a wide range of mental disorders, including depression and anxiety, and also in helping prevent recurrence [21]. In addition to face-to-face CBT, videoconference-delivered CBT (vCBT) is known to be effective in treating many mental disorders. Videoconferencing systems transmit audio and video signals over the internet and are used in treating mental health problems [22-24]. Research has demonstrated the effectiveness of vCBT for anxiety disorders [25] and chronic pain [26].

In this study, we administered CBT to the parents of adolescents with internet addiction, based on Community Reinforcement and Family Training (CRAFT) [27-29], a cognitive-behavioral program designed to get individuals who abuse substances and refuse treatment to enter treatment by teaching family members how to support a clean or sober lifestyle [30-33]. In Japan, CRAFT has also been used for families of youth in withdrawal [34].

This study aimed to conduct a pilot randomized controlled trial (RCT) to examine the feasibility of vCBT for parents of adolescents with internet addiction. Questionnaires and other surveys were administered to adolescents with internet addiction; however, the adolescents themselves did not receive any direct interventions in the form of vCBT.

## Methods

### Participants

The inclusion criteria of adolescents with internet addiction and their parents were as follows: (1) parents of adolescents with internet addiction (aged 12-20 years at the time of the survey) who provided consent; (2) parents who were aged at least 20 years and living with the adolescent with internet addiction at the time of providing consent; (3) adolescents and parents who were adequately informed, fully understood, and had given their free and voluntary written consent to participate in this study; (4) adolescents who were able to complete the self-administered questionnaire; (5) adolescents with internet addiction who scored 40 or higher on the self-administered Internet Addiction Test (IAT); and parents who were healthy enough to receive continuous CBT counseling support once a week for 12 weeks at home, using a videoconferencing system, and who had no physical or mental illness or disability that would interfere with counseling support.

Exclusion criteria for adolescents with internet addiction and their parents were as follows: (1) those who had difficulty obtaining ongoing counseling support due to problem behaviors, such as severe self-injurious behaviors, including cutting wrists or experiencing domestic violence; (2) those who had complications, such as a severe psychotic disorder, bipolar disorder, drug dependence, or alcohol-dependence, that are likely to interrupt CBT due to worsening of these symptoms; (3) those who were at an imminent risk of suicide, exhibited repeated antisocial behavior, or had progressive illness, thereby consequently discontinuing CBT; (4) those who had difficulty contacting the investigator; and (5) those deemed by the principal investigator or subinvestigator to be unfit for the safe conduct of this study.

Participants were recruited through posters on the Chiba University website; the Chiba University Hospital and its affiliated hospitals; and educational institutions in the Chiba Prefecture in Japan. During enrollment, adequate written and oral explanations were provided to the adolescents and their guardians. Only those who provided written and oral consent of their own free will were enrolled. However, if it were difficult for the participants to visit Chiba University (eg, because of COVID-19), an initial diagnostic evaluation was conducted remotely via videoconferencing.

### Randomization

Parents of adolescents with internet addiction who agreed to the study design were asked to come to the hospital once—for the diagnostic evaluation; they were examined directly by the physician, and then divided, by random assignment, into a vCBT group (research treatment intervention group) and a waitlist control group. Participants who were unable to visit Chiba University Hospital due to COVID-19 restrictions were offered the option of completing the web-based initial diagnostic assessment for this study (to determine their eligibility to receive CBT counseling), so that they could participate in the clinical trial as soon as possible.

The case registration system was centrally administered at the Clinical Research Data Center (Department of Clinical Trials, Chiba University Hospital). The case registration was performed by issuing subject identification codes in the electronic medical record using the clinical trial system, creating a correspondence table, and performing linkable anonymization. For case registration, subject identification codes were issued, correspondence tables were created, and linkable anonymization was performed. The screening period was no longer than 8 weeks.

### Sample Size

Considering this was a pilot study, the target number was calculated to ensure that the safety and feasibility of conducting a large-scale RCT in the future could be verified. Subsequently, 20 patients were targeted for analysis (CBT group: n=10, 50%; waiting list group: n=10, 50%). The main hypothesis of this study was to verify the superiority of the symptom-improving effect of the combined CBT group on patients with internet addiction.

### Study Design

This study was an RCT (2 parallel groups) and a prospective intervention study. As mentioned above, parents were randomly assigned to either the vCBT group (the intervention group) or the control group.

In the vCBT group, for each parent, sessions were held weekly for 50 minutes each, over 12 weeks, either remotely or in person. The primary efficacy and safety outcomes were assessed before the intervention began (week 0), at the end of the intervention (week 13), and during the follow-up period (week 24).

In the control group, the parents and adolescents waited without intervention. The primary efficacy and safety outcomes were assessed only before the start of the intervention (week 0) and at the end of the intervention (week 13), but not during the follow-up period (week 24).

### Intervention: vCBT for Parents

As mentioned above, CBT was administered to the parents of adolescents with internet addiction, based on CRAFT [23,24]. The program was designed to reduce the burden on family members of addicted individuals, improve family relationships, and promote the use of medical treatment services for individuals with addiction. The content and objectives of all 12 sessions are listed in Table 1 [27,29-33].

Sessions were held weekly for 50 minutes each over 12 weeks focusing on a specific theme. Parents completed the homework presented after the session and aimed to solve their communication problems with their adolescents. In the waiting list control group, parents and adolescents waited without receiving any intervention. Tests related to the primary outcome for evaluating efficacy and safety were to be conducted only before the start of the intervention (week 0) and at the end of the intervention (week 13), but not during the follow-up period (week 24). The waiting list control group then received CBT similar to the vCBT group.

**Table 1:** Content of each session.

Times	Contents	Purpose
1	Assessment interview	Conduct specific interviews with the adolescent with internet addiction about their daily life and set treatment goals.
2	What is internet dependence? (psychoeducation)	Parents gain a better understanding of internet addiction and cognitive behavioral therapy.
3	Understanding problematic behaviors	Categorize the adolescent’s desirable and undesirable behaviors in daily life.
4	Case formulation (functional analysis)	Parents learn how to conduct a functional analysis of problem behaviors and recognize external and internal triggers.
5	CRAFT^a^ (problem solving)	Parents understand the CRAFT problem-solving method. They also practice the RIBEYE^b^ and score evaluation methods. RIBEYE is one of the problem-solving techniques in CRAFT.
6	CRAFT (assertion)	Encourage parents to acquire positive communication skills. Additionally, parents undergo assertion training.
7	Effective praise	Parents learn strategies to encourage positive behaviors in adolescents with internet addiction, while also addressing their own actions and taking a proactive approach to reduce negative behaviors.
8	What the family does first?	In order to enrich the lives of the whole family, parents reconsider their roles and rebuild their relationships with their children.
9	How to treat children?	Parents understand “6 ways to protect adolescents from internet addiction.”
10	Stability of the whole family	Parents understand ways to prevent violent behavior and learn the importance of improving the whole family's overall stability to help address the problem of their adolescent’s internet addiction.
11	Treatment of internet addiction	Review and generalize previous treatment sessions.
12	Relapse prevention	Approach appropriate counseling institutions.

^a^CRAFT: Community Reinforcement and Family Training.

^b^RIBEYE: relax, identify, brainstorm, evaluate, yes to one, encourage yourself. This is a method in which the therapist (supporter) helps the client solve the problems they are facing. It is said that by mastering this method, the range of thinking will be broadened, and the client will be able to respond calmly and flexibly.

### Outcomes

#### Primary Outcome: Young IAT

The primary outcome was the scores of the Young IAT [34] reported by the adolescents. The 20 items were rated on a 5-point Likert scale, ranging from 1=not at all to 5=frequently, to indicate the extent to which internet use interfered with their daily life, family relationships, social life, personal health, and state of mind. The score ranged from 20 to 100, with higher scores indicating greater problems caused by internet use. Young IAT defines a score between 20 and 49 as an average user who is in control of their internet use, a score of 50 to 79 as an addicted user with occasional or frequent problems with their internet use, and a score of 80 to 100 as an addicted user with major problems with their internet use.

For adolescents with internet addiction, the primary outcome was the change in IAT scores at week 13, compared to week 0 (baseline).

#### Secondary Outcomes

For adolescents with internet addiction, the following secondary outcomes were included to measure the effectiveness of the intervention: (1) daily hours of internet use as reported by adolescents; and (2) the Japanese version of the EQ-5D-5L [35-39] questionnaire, a self-reported quality-of-life index.

##### EQ-5D-5L Questionnaire

The EQ-5D-5L is a self-management questionnaire and rating system comprising 5 dimensions (mobility, self-care, usual activities, pain or discomfort, and anxiety or depression), each with 5 levels of severity (no problems, mild problems, moderate problems, severe problems, and extreme problems). The 5 dimensions of responses can be combined into a 5-digit number describing the health status of the respondent (11111=no problem at all and 55555=extreme problems). This defines a total of 3125 possible health states, which can be converted into a single health index by applying a formula that assigns a value to each response. To obtain the EQ-5D-5L index, the Japanese version of the EQ-5D-5L was used.

For the parents, the secondary outcomes included: (1) daily hours of internet use by adolescents as reported by parents; (2) parent-administered Japanese version of the EQ-5D-5L; and (3) Young Diagnostic Questionnaire (YDQ).

##### YDQ Instrument

To assess problematic internet use, the parents answered the YDQ [40,41]. The YDQ consists of 8 items (binary response format: 0=no, 1=yes). By summing up the values of all 8 items of the questionnaire, a YDQ sum score (range 0-8) was calculated with a higher sum indicating a higher risk of problematic internet use among adolescents.

### Ethical Considerations

The Research Ethics Committee of Chiba University Hospital approved this study (IRB: 607). Written informed consent was obtained from all participants. Data were anonymized and participants in this study were not compensated. The study registration is UMIN000032483. This study was funded by Grants-in-Aid for Scientific Research (C) 20K10350.

### Statistical Analysis

The distribution and summary statistics of subject background data were calculated in each analysis. For nominal variables, the frequency and proportion of categories are shown for each group. For continuous variables, summary statistics (number of cases, mean, SD, minimum, median, and maximum) were calculated for each group. Comparisons between groups were performed using the Pearson chi-square test for nominal variables, the Fisher exact probability test when more than 20% of cells had expected frequencies fewer than 5, and 2-tailed *t* tests for continuous variables. The significance level was set at 5%.

For the primary outcome, the adolescents’ IAT score at the start of the study was used as the baseline value, and statistical analyses were performed to determine the change at the end of treatment. The 2-sided significance level for hypothesis testing was set at the 5% level, and the 2-sided 95% CIs were calculated. The primary objective of the study was to determine whether the vCBT group showed significant improvement over the control group at week 13, based on the adolescents’ IAT scores. The null hypothesis posited no significant difference in the changes of IAT scores between the 2 groups in the primary analysis, which was tested using analysis of covariance. The covariates included in this analysis were factors used for allocation adjustment: sex, age less than 16 years, and age 16 years and older [34]. To complement the results of the primary analysis, secondary outcomes, briefly described here, were analyzed using both analysis of covariance and linear mixed-effects models, without adjustment for multiplicity. The significance level for hypothesis testing was set at a 2-sided 5%, and corresponding 2-sided 95% CIs were calculated. All statistical analyses were executed using R (version 4.2.1, R Foundation for Statistical Computing), with a significance level set at *P*<.05.

## Results

### Participants

**Figure 1:**
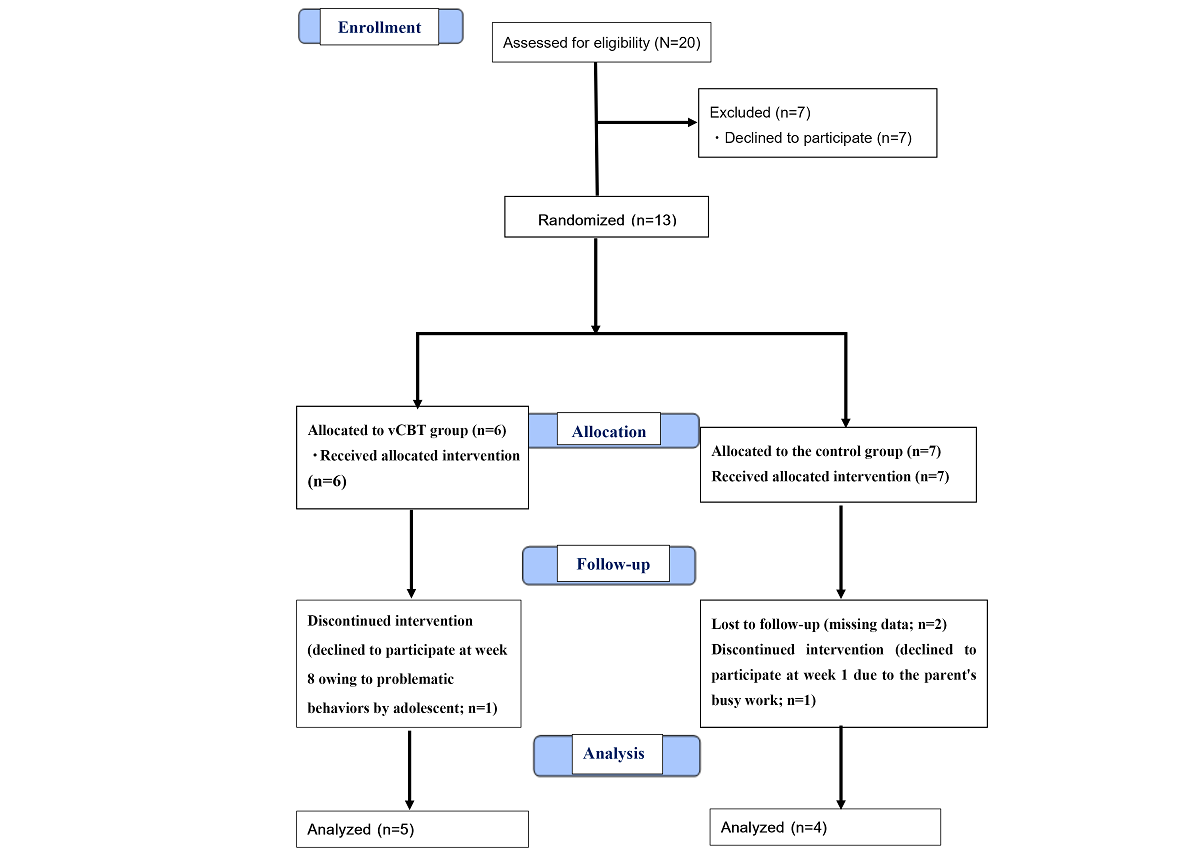
Flow diagram of the participant selection process based on Consolidated Standards of Reporting Trials (CONSORT) guidelines. vCBT: videoconference-delivered cognitive behavioral therapy.

The study period was from March 1, 2018, to March 31, 2022. Figure 1 shows the characteristics of the participants. Of the 20 participants who were assessed for eligibility, 7 declined to participate. Finally, 13 participants and their adolescents (vCBT group: n=6, 46%; control group: n=7, 54%) provided informed consent, and were randomized to receive the intervention (Multimedia Appendix 1).

During the follow-up, 1 patient in the vCBT group discontinued the intervention. This youth withdrew from participation in week 8 due to behavioral problems. In the control group, 2 patients were lost to follow-up due to missing data, and 1 patient discontinued the intervention because their parents refused to participate in the first week due to work reasons. Finally, 5 patients in the vCBT group and 4 in the control group were analyzed.

### Adverse Events

One of the participants in the intervention group declined to participate in this study at week 8 due to her adolescent’s problematic behaviors. After that, the problem behavior disappeared. Table 2 shows the characteristics of participants. There were 0 male and 6 female participants in the vCBT group and 1 male and 6 female participants in the control group. The mean age of the participants was 47.33 (SD 4.6) years in the vCBT group and 50.29 (SD 5.2) years in the control group. As for adolescents, 5 were male and 1 was female in the vCBT group and 6 were male and 1 was female in the control group. The mean age of the adolescents was 15.00 (SD 2.2) years in the vCBT group and 15.57 (SD 2.1) years in the control group.

**Table 2:** Characteristics of the participants.

Participant and characteristics				vCBT^a^ group (n=6)		Control group (n=7)		*P* value^b^
**Parent**								
	**Sex, n (%)**							
		Male	0 (0)		1 (14)		≥.99	
		Female	6 (100)		6 (86)		≥.99	
	**Age (years), mean (SD)**		47.33 (4.6)		50.29 (5.2)		.30	
**Adolescent**								
	**Sex, n (%)**							
		Male	5 (83)		6 (86)		≥.99	
		Female	1 (17)		1 (14)		≥.99	
	**Age (years), mean (SD**)		15.00 (2.2)		15.57 (2.1)		.64	

^a^vCBT: videoconference-delivered cognitive behavioral therapy.

^b^2-tailed *t* test.

### Primary Outcome

The primary outcomes are shown in Table 3. As assessed by the IAT for youths, the vCBT group scored 67.7 (SD 18.3; 6/13, 46%) points and the control group 66.9 (SD 21.9; 7/13, 54%) points at week 0. At week 13, the vCBT group scored 56.2 (SD 25.1; 5/9, 56%) points and the control group scored 68.0 (SD 18.7; 4/9, 44%) points. The change from baseline was –7.80 (SD 17.3) points in the vCBT group and 1.75 (SD 16.6) points in the control group. There was no significant change of IAT between the 2 groups (*P*=.43; Table 3).

**Table 3:** Primary outcome.

Time point and outcome		vCBT^a^ group	Control group	*P* value^b^		*P* value^c^
**Week 0 (n=13)**					.94	N/A^d^
	Participants, n (%)	6 (46)	7 (54)			
	Internet Addiction Test score, mean (SD)	67.7 (18.3)	66.9 (21.9)			
**Week 13 (n=9)**					.46	N/A
	Participants, n (%)	5 (56)	4 (44)			
	Internet Addiction Test score, mean (SD)	56.2 (25.1)	68.0 (18.7)			
**Change** **from baseline**					.43	.57
	Participants, n (%)	5 (56)	4 (44)			
	Internet Addiction Test score, mean (SD)	–7.80 (17.3)	1.75 (16.6)			

^a^vCBT: videoconference-delivered cognitive behavioral therapy.

^b^2-tailed *t* test.

^c^ANCOVA.

^d^N/A: not applicable.

### Secondary Outcomes

As shown in Table 4, the change from baseline in adolescents’ hours of internet use per day was –2.30 (SD 1.86) hours for the vCBT group and –0.50 (SD 1.00) hours for the control group. There was no significant difference of change in internet use time reported by the adolescents between the 2 groups (*P*=.33).

**Table 4:** Secondary outcomes.

Change from baseline	vCBT^a^ group (n=5)	Control group (n=4)	*P* value^b^	*P* value^c^
Internet usage time (hours) reported by adolescents, mean (SD)	–2.30 (1.86)	–0.50 (1.00)	.33	.71
Adolescent’s EQ-5D-5L index score (QOL^d^), mean (SD)	0.04 (0.28)	0.06 (0.18)	.45	.38
Internet usage time (hours) reported by parent, mean (SD)	–2.00 (3.81)	0.25 (1.26)	.14	.84
Parent’s EQ-5D-5L index score (QOL), mean (SD)	0.20 (0.30)	–0.06 (0.18)	.01	.84
Parent’s Young Diagnostic Questionnaire scores, mean (SD)	–0.20 (1.30)	0.50 (1.73)	.70	.09

^a^vCBT: videoconference-delivered cognitive behavioral therapy.

^b^2-tailed *t* test.

^c^ANCOVA.

^d^QOL: quality of life.

For the EQ-5D-5L in adolescents, the change from baseline was 0.04 (SD 0.28) points for the vCBT group and 0.06 (SD 0.18) points for the control group. There was no significant change in the QOL of the adolescents between the 2 groups (*P*=.45).

The parent-reported change from baseline in the adolescents’ daily internet use time was –2.00 (SD 3.81) hours for the vCBT group and 0.25 (SD 1.26) hours for the control group. There was no significant difference of internet use time reported by the parents between the 2 groups (*P*=.14).

For the EQ-5D-5L, a parent QOL index, the change from baseline was 0.20 (SD 0.30) points for the vCBT group and –0.06 (SD 0.18) points for the control group. The change of QOL of the parents in the vCBT group was significantly higher than that in the control group (*P*=.01).

For the YDQ, the change from baseline was –0.20 (SD 1.30) points for the vCBT group and 0.50 (SD 1.73) points for the control group. There was no significant difference in change of YDQ between the 2 groups (*P*=.70).

## Discussion

### Overview

We conducted vCBT for parents of adolescents with internet addiction and tested its effectiveness in improving the symptoms of internet addiction in adolescents. In this study, the change in the IAT from the start of intervention week 0 to the end of intervention week 13 was –11.5, from 67.7 (6/13, 46%) to 56.2 (5/9, 56%) in the vCBT group, and 1.1, from 66.9 (7/13, 54%) to 68.0 (4/9, 44%) in the control group. We did not find a statistically significant difference between the vCBT group and the control group in this study. In the completed analysis, the change in the IAT in the vCBT group was –7.8, from 64.0 (5/9, 56%) to 56.2 (5/9, 56%), and the change in the IAT in the control group was 1.75, from 66.3 (4/9, 44%) to 68.0 (4/9, 44%).

A systematic review of meta-analyses of treatments for internet addiction shows that CBT is generally effective [42,43]. For the primary outcome, Bernstein et al [44] conducted a 2-arm RCT of a digital health intervention in 130 people with internet use disorders. An intervention group of 65 participants received 7 of their CBT-based sessions and were compared to a waitlist control group of 65. The results showed the IAT of the intervention group decreased by 7.99, from 63.46 to 55.47, and the waitlist control group decreased by 3.09, from 63.89 to 60.8. This was statistically significant (*d*=0.54, 95% CI 0.19-0.89).

Similarly, Yang et al [45] conducted an RCT comparing an intervention group and a control group to examine the effects of a short-term intensive CBT intervention on internet addiction among Chinese university students. Intensive CBT comprised 5 sessions of 90 minutes each, totaling 7.5 hours. The intervention group received an intervention program in addition to an internet addiction training course. By contrast, the control group received only an internet addiction training course. Consequently, from baseline to posttreatment assessment, the IAT decreased by 7.3 from 59.6 to 52.3 in the intervention group, and by 1.1 from 59.9 to 58.8 in the control group.

Compared to the studies by Bernstein et al [44] and Yang et al [45], our study showed similar reductions in the CBT intervention group. However, these studies directly intervened with individual internet addicts, whereas our study differed in that the target of intervention was the parents of adolescents with internet addiction.

For the secondary outcomes, regarding the amount of time adolescents use the internet per day, Wölfling et al [46] conducted a 2-arm RCT of a CBT called short-term treatment for internet and computer game addiction; it consisted of 15 weekly group and up to eight 2-week individual sessions in 143 people with internet addiction, compared to a waitlist control group of 71. As a result, the amount of time spent on the internet a weekday decreased by 3.1 hours for the intervention group, from 6.5 to 3.4 hours and remained unchanged for the waitlist control group at 5.8 hours. The amount of time spent on the internet on weekends decreased for the intervention group by 4.3 hours, from 8.4 to 4.1 hours, and for the waitlist control group by 2.0 hours, from 7.6 to 5.6 hours. They showed that the intervention group was statistically significant on both weekdays and weekends (*P*≤.001).

In this study, the amount of change in youth-reported internet usage time in the intervention group decreased by 2.30 hours, from 11.4 to 9.1 hours, and that in the control group by 0.50 hours, from 9.25 to 8.75 hours (*P*=.33). Although our study did not distinguish between weekdays and weekends, the values in Wölfling et al [46] study and ours were similar. Taken together, these results suggest that CBT may help patients with internet addiction reduce internet usage time.

Zhu et al [47] conducted a systematic review of 3538 internet addicts and a total of 57 RCTs to examine the effectiveness, benefits, and drawbacks of various treatments used alone or in combination. Their network meta-analysis of 13 interventions showed that the top 4 were repetitive transcranial magnetic stimulation+CBT, drugs+other, repetitive transcranial magnetic stimulation, and electroacupuncture+CBT. Cañas and Estévez [48] conducted a systematic review of intervention or prevention programs for excessive internet use among adolescents and considered 14 programs that met inclusion criteria. Both systematic review studies were regarding interventions for internet-addicted adolescents themselves; no study about CBT for parents of adolescents with internet addiction was identified.

Concerning family therapy as the intervention, Liu et al [49] conducted multifamily group therapy for each family with an adolescent with internet addiction (aged 12-18 years) and a parent (aged 35-46 years). They showed that the intervention group was significantly better than the control group on the adolescent pathological internet use scale, the internet use time, the parent-child communication scale, and the parent-child intimacy scale.

In addition to the previous studies about CBT for adolescents with internet addiction themselves or family therapy, this study suggested the feasibility of vCBT for parents of adolescents with internet addiction.

### Limitations

A few limitations of this study must be acknowledged. First, although the implementation plan called for 20 participants (vCBT group: n=10, 50%; control group: n=10, 50%), the final analysis included a smaller number of 9 respondents (vCBT group: n=5, 56%); control group: n=4, 44%). The sample size for this study was examined to determine what sample size would be needed to show a significant difference; based on the results of this exploratory RCT, statistical analysis indicated that the ideal sample size would be 108 cases for vCBT (n=54) and control group (n=54) combined. This was substantially larger than the current sample size.

Second, the waiting list group was used as a control group because of ethical reasons. As the waitlist control may be inappropriate for evaluating treatment outcomes, alternative treatments should be used as a control group in future large-scale RCTs. Third, this was a single-center RCT and not a multicenter study, which affects its generalizability. Fourth, since there are no data on follow-up after the vCBT intervention in this study, future efforts should focus on collecting follow-up data and implementing efficient strategies to ensure a higher response rate. Fifth, for the primary outcome, we used the Young IAT. In the systematic review by meta-analysis, the IAT was the most used measure of internet addiction; however, other measures should be considered as well. Specifically, data should be collected by objective machines, such as tablets, on the time spent on the internet by addicts. Additionally, other evaluation measures that have emerged since the IAT, such as the Compulsive Internet Use Scale, should be examined [50,51].

### Conclusions

This study suggested the practical feasibility of vCBT for parents of adolescents with internet addiction. Further large-scale, multicenter RCT is necessary to examine the effectiveness.

## Appendix

Multimedia Appendix 1 Analysis results by subject background.

Multimedia Appendix 2 CONSORT‐eHEALTH checklist (V.1.6.1).

## Abbreviations

CBT: cognitive behavioral therapy
CRAFT: Community Reinforcement and Family Training
IAT: Internet Addiction Test
QOL: quality of life
RCT: randomized controlled trial
vCBT: videoconference-delivered cognitive behavioral therapy
YDQ: Young Diagnostic Questionnaire
