# Supplementary figures and images for: Videoconference-Delivered Cognitive Behavioral Therapy for Parents of Adolescents With Internet Addiction: Pilot Randomized Controlled Trial
Source: JMIR Pediatr Parent. 2024 Oct 3;7:e60604. doi: 10.2196/60604 (PMC11487207; doi:10.2196/60604)

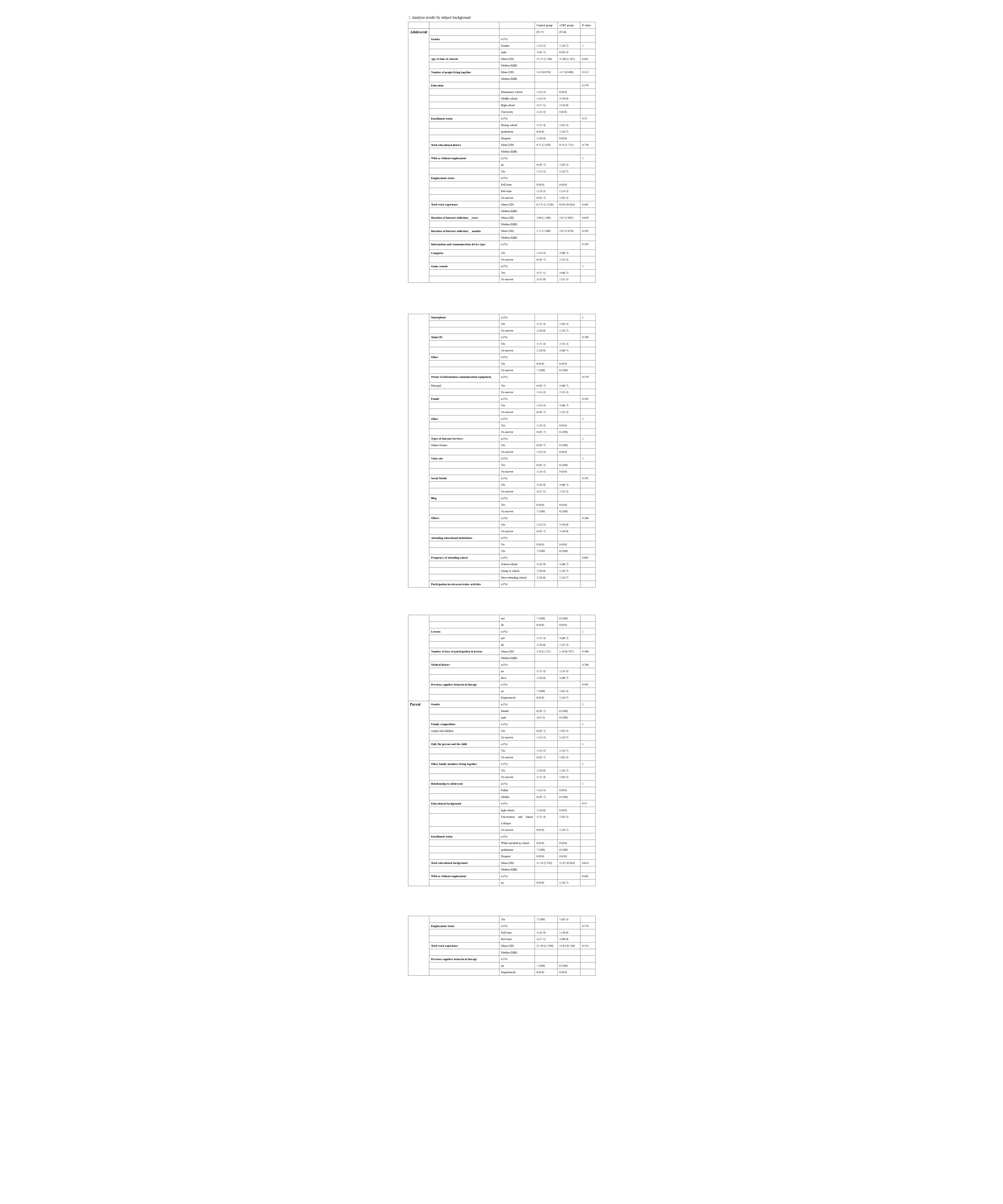

Supplement: Multimedia Appendix 1 [file pediatrics_v7i1e60604_app1.png]
